# The effects of instability core training on balance ability and paddling performance among young male Chinese flatwater sprint kayakers: a randomized controlled trial

**DOI:** 10.1186/s13102-025-01248-6

**Published:** 2025-07-10

**Authors:** Jianxin Gao, Zhigang Gong, Shamsulariffin Samsudin, Borhannudin Bin Abdullah, Roxana Dev Omar Dev

**Affiliations:** 1 College of Physical Culture, Jiangxi Teachers College, Yingtan, Jiangxi 335000 China; 2 Key Lab of Aquatic Sports Training Monitoring and Intervention of the General Administration of Sport of China, Faculty of Physical Education, Jiangxi Normal University, Nanchang, 330000 China; 3 Department of Sport Studies, Faculty of Educational Studies, University Putra Malaysia, Serdang, Selangor 43400 Malaysia

**Keywords:** Instability core training, Unstable surface, Balance ability, Paddling performance, Kayakers

## Abstract

**Background:**

Instability core training (ICT) has been widely used in various sports as a training method to enhance athletes' balance ability and athletic performance. The study aimed to examine the impact of ICT performed on unstable surfaces (BOSU balls, Swiss balls, and Wobble boards) versus traditional core training (TCT) performed on stable surfaces (floor and bench) on balance ability and paddling performance among young male Chinese kayakers.

**Method:**

A randomized controlled trial (RCT) recruited 63 eligible kayakers aged 16–19 years from the Nanchang Yao Lake kayaking training base in Jiangxi province, China. Participants were randomly assigned to the ICT group and the TCT group. Both groups completed a 12-week core training program consisting of 1-h sessions, 3 times/week. Static balance ability was assessed using the Flamingo Balance Test (FBT), while dynamic balance ability was measured using the Star Excursion Balance Test (SEBT). Paddling parameters were evaluated using the average stroke power and stroke rate for the men’s K-1 200 m land dynamometer/ergometer sprint tests. Statistical analyses were conducted via multivariate analysis of variance (MANOVA), with the significance level set at *P* < 0.05.

**Results:**

The analysis for within-group effects demonstrated statistically significant improvements in static balance ability, dynamic balance ability, average stroke power, and stroke rate variables between the pre-test and post-test in both the ICT and TCT groups (*p* < 0.05). No statistically significant differences were observed in the pre-test (*p* > 0.05) for between-group effects. In contrast, statistically significant differences were found between the ICT and TCT groups in the post-test for all balance ability and paddling parameter variables (*p* < 0.05).

**Conclusion:**

The findings suggest that while TCT significantly improves balance ability and paddling performance, ICT is more effective than TCT over a 12-week intervention among young male Chinese kayakers. Therefore, it can replace TCT, as it promotes better improvement in balance ability and paddling parameters for young male Chinese kayakers.

Trial registration.

The full name of the registry: Effect of instability resistance training on balance, core muscle strength, and athletic performance. The trial registration number is NCT06432595. The date of registration is 07/01/2024. The trial registration platform is ClinicalTrials.gov PRS (https://clinicaltrials.gov/).

## Introduction

Flatwater sprint kayaking is a highly dynamic and competitive water sport that demands athletes to generate maximum strength, speed, and endurance within a short timeframe [1, 2]. Performance in flatwater sprint kayaking is primarily determined by race time, which depends on the average velocity maintained over a given distance [3, 4]. In short-distance events such as the 200 m sprint, elite male kayakers typically complete the race in 34—40 s, achieving an average speed of 4.95—5.88 m/s [5]. Success in these races requires athletes to maximize power output while maintaining technical efficiency to minimize fatigue and sustain peak speed [6]. The race is divided into four key phases—preparation/start, acceleration, midcourse, and final sprint—during which athletes must execute high-intensity alternating paddling or strokes to propel the kayak forward [7, 8].

In sprint kayaking, paddling force (stroke power) and paddling frequency (stroke rate) are critical determinants of performance [7]. A higher average paddling force enables more excellent propulsion per stroke, which is particularly important in short-distance events requiring rapid acceleration. Studies have shown that kayakers generate peak paddling forces during the start and final sprint phases to achieve and sustain top speed [9]. Simultaneously, a high paddling frequency allows for greater stroke efficiency, enabling kayakers to maintain velocity. Elite kayakers strategically modulate their stroke rate, employing higher frequencies during acceleration and sprint phases to optimize performance [10]. Specifically, world-class male kayakers in 200 m sprints generate peak average paddle forces of 400–600 *N* while maintaining stroke rates of 120–140 strokes per minute to maximize propulsion and efficiency [8, 11].

Given the inherently unstable nature of kayaking, balance ability plays a fundamental role in optimizing paddling performance. Balance ability refers to the body's capacity to maintain postural stability and adjust to external disturbances [12, 13]. It is categorized into static balance ability, essential for postural control and force transfer, and dynamic balance ability, crucial for coordinating movements and generating propulsion [14, 15]. Kayaking paddling performance is influenced by uncontrollable factors such as water currents, wind conditions, and environmental variables [16, 17]. Additionally, the kayaker’s body experiences multidirectional oscillations during the paddling phases, with upper body instability particularly pronounced, including trunk flexion, extension, and rotation [18]. Excessive body unstable movement can disrupt the paddling phase, alter stroke mechanics, and negatively impact sprint performance [19, 20]. Moreover, data from cross-sectional studies indicated significant relationships between balance ability, stroke power, and stroke rate in kayakers [5, 21, 22]. Thus, since kayaking is performed in a seated position on an unstable water surface, athletes must develop superior static and dynamic balance abilities to stabilize posture and optimize stroke execution.

A widely recognized approach for improving balance ability is instability core training (ICT) [23–25]. Granacher et al. (2014) [26] categorized core training methods into two main types based on support surfaces and external conditions: traditional core training (TCT) and instability core training (ICT). TCT is typically performed on stable surfaces such as the floor or a bench [27, 28], whereas ICT incorporates various unstable environments, including surfaces like water, sand, and gravel, as well as equipment such as Swiss balls, BOSU balls, foam shafts, and balance boards. Additionally, specialized unstable training tools like elastic bands, suspension chains, and ropes are often utilized to challenge the core further [29, 30]. Although both TCT and ICT target similar muscle groups, they elicit distinct neuromuscular and sensorimotor adaptations due to differences in training stimuli. ICT, by involving unstable surfaces, increases the demand for proprioceptive feedback, postural adjustments, and reflexive core activation [24]. These stimuli promote heightened activation of deep stabilizing muscles such as the transverse abdominis, multifidus, and pelvic floor muscles, which are essential for spinal control and intersegmental stability [25]. In contrast, TCT often favors the development of gross motor strength and superficial musculature through isotonic contraction under stable conditions. Moreover, ICT is associated with enhanced sensorimotor integration, particularly involving the vestibular, visual, and somatosensory systems, leading to improved balance and motor coordination [23]. Studies using electromyography (EMG) and balance tests have demonstrated that ICT leads to superior activation of trunk stabilizers and faster motor unit recruitment compared to TCT [5, 21]. This heightened neuromuscular control is especially beneficial in unstable sport-specific environments like kayaking, where athletes must make rapid postural corrections in response to unpredictable perturbations from the water surface. Research has also consistently shown that although both TCT and ICT involve similar core exercises, they induce different neuromuscular adaptations due to their distinct physiological mechanisms in improving balance ability [31–36]. Furthermore, ICT has been found to significantly enhance balance ability across a wide range of sports, including soccer [37–42], basketball [43, 44], handball [45, 46], weightlifting [47, 48], and volleyball combined with soccer [49, 50]. Similar improvements have been observed in other disciplines such as judo [51], archery [52], rhythmic gymnastics [53], badminton [54], as well as sprinting [55] and gymnastics [56].

Although instability core training (ICT) has demonstrated its effectiveness in improving balance ability across various sports, its specific impact on paddling performance in kayakers, particularly young male Chinese kayakers, remains insufficiently explored. In addition, adolescence is a critical developmental period during which athletes experience significant increases in growth and sex hormone secretion, leading to notable improvements in the vestibular, visual, and proprioception systems for balance ability [57]. However, international competition statistics reveal significant gaps in both balance ability and paddling performance between young male Chinese kayakers and their world-class counterparts [58–62]. Therefore, the discrepancy among young male Chinese kayakers may be partly attributed to the lack of innovative core training methods tailored to the specific instability characteristics of kayaking, particularly in adapting to dynamic, unpredictable water conditions [63].

According to the principle of training specificity, practical training should closely replicate the movement demands of the sport [23]. Considering the inherent instability challenges in kayaking, integrating unstable elements into core training could offer a valuable stimulus for improving balance ability and paddling performance in young Chinese kayakers. Thus, this study seeks to examine the comparative effects of instability core training (ICT) versus traditional core training (TCT) on balance ability and paddling performance, aiming to bridge the existing research gap and provide evidence-based recommendations for enhancing core training strategies in flatwater sprint kayaking for young male Chinese kayakers.

## Methods

### Participants

This study adhered to the CONSORT guidelines and employed a randomized controlled trial (RCT) design [64]. The required sample size was determined using G*Power 3.1 software, based on a repeated-measures design involving two groups and two time points. Sample size estimation was performed using an F-test family: MANOVA for repeated measures (within-between interaction). To ensure sufficient statistical power to detect meaningful differences between groups, even in the presence of small effects, the calculation was based on the smallest expected effect size reported in a systematic review of instability core training (ICT) versus traditional core training (TCT) interventions in youth athletes [65]. Specifically, an effect size (f) = 0.2 was selected, corresponding to changes in static balance ability assessed by the Stork Balance Test [38]. Additionally, the α level of 0.05 is commonly used in behavioral and scientific research to limit the probability of falsely detecting an effect (Type I error), while the statistical power level of 0.80 ensures an 80% chance of correctly detecting a true effect [66]. Therefore, input parameters included a Type I error rate (α) = 0.05, statistical power (1-β) = 0.80, two groups, and two measurement points. Based on these assumptions, the minimum required total sample size was 52 participants (26 per group). To account for a potential 10% dropout rate [67], the final adjusted sample size was increased to 58 participants (29 per group), ensuring adequate power for all planned analyses.

A total of 243 young male kayakers from the Nanchang Yao Lake kayaking training base were screened for eligibility. After applying the inclusion and exclusion criteria, 96 kayakers were deemed eligible for participation. The inclusion criteria were as follows: (1) male kayakers specializing in the 200 m event, aged 16—19 years; (2) a minimum of three years of kayak-specific training; (3) no history of surgery, health issues or recent injuries; and (4) no prior systematic training in ICT. The exclusion criteria were: (1) recent sports injuries or health issues, and (2) current participation in an ICT program. However, 27 athletes declined to participate due to academic commitments, such as high school and college entrance exams, while six could not participate due to parental objections.

The selection of young male kayakers as participants was intentional to control for potential confounding factors related to sex and age, which can influence neuromuscular responses and adaptations to core training interventions. Males and females may differ in trunk muscle activation patterns, hormonal responses, and balance strategies, which could introduce variability and complicate the interpretation of training effects. By focusing on a homogeneous sample, the study aimed to isolate the impact of the ICT intervention more precisely. However, this approach does limit the generalizability of the findings. Future research should include female athletes and athletes across different age groups to better understand sex- and age-related differences in training responsiveness within the context of sprint kayaking.

**Fig. 1:**
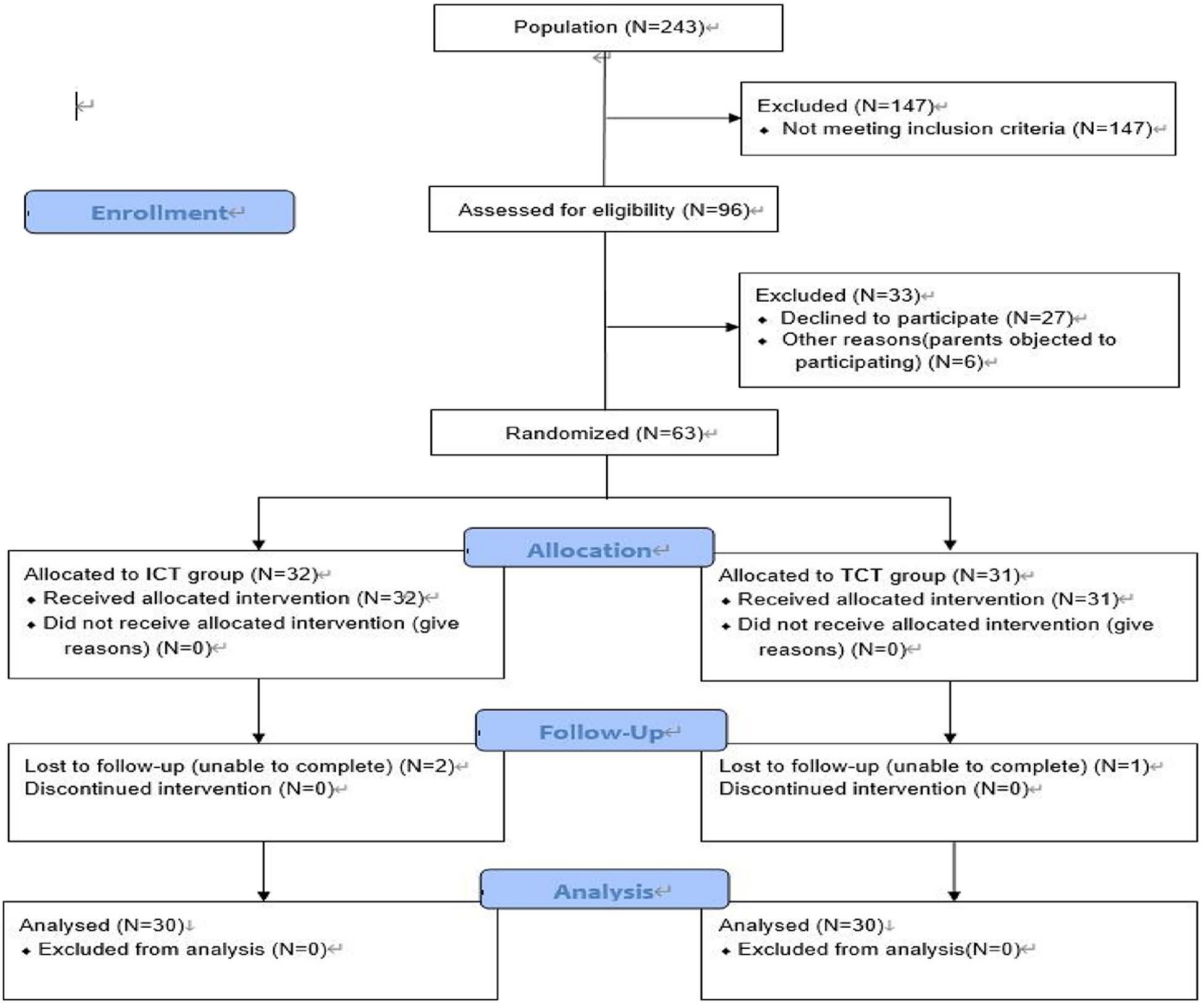
CONSORT flow diagram

Ultimately, 63 kayakers (the baseline on demographic and anthropometric characteristic variables for subjects see Table 1) voluntarily participated and were randomly assigned to either the ICT group (*N* = 32) or the TCT group (*N* = 31) with the Lottery method. Written informed consent was obtained from all participants, co-signed by a parent or guardian. Finally, in the ICT experimental group, 2 participants dropped out because they could not complete the entire training program due to participation in the national kayaking competition. The total dropout ratio was 6.25%. In the TCT control group, 1 participant dropped out of the test due to participation in the national kayaking competition. The total dropout ratio was 3.22%. Therefore, the ICT and TCT groups had 30 participants, respectively, and each young male kayaker who completed the training protocol was available for final analysis. For the final statistical analysis, there were a total of 30 valid data points: ICT (*N* = 30) and TCT (*N* = 30). The study received ethical approval from the Ethics Committee of Universiti Putra Malaysia (Approval No. JKEUPM 2023–256), and all participants provided informed consent before participation. Figure 1 presents the study protocol and participant flow (see Fig. 1).

**Table 1:** The baseline on demographic and anthropometric characteristic variables for subjects

Variables	ICT group (*N*=30)	TCT group (*N*=30)
Age (years)	18.06±1.20	17.43±1.35
Height (cm)	177.11±7.80	179.33±7.34
Weight (kg)	71.33±10.79	73.40±8.67
Training years (years)	4.23±1.27	3.83±1.17

*ICT* Instability *Core* Training, *TCT* Traditional Core Training

### Intervention

This study's core training protocol involved two approaches: instability core training (ICT) for the experimental group and traditional core training (TCT) for the control group. Both groups followed a structured 12-week program divided into three progressive phases: primary (weeks 1–4), intermediate (weeks 5–8), and advanced (weeks 9–12). Each phase included the same six core exercises—three static (Prone Plank, Shoulder Bridge, and Lateral Bridge) and three dynamic (Crunch, Back Extension, and Russian Twist) (see Table 2). The primary distinction between the two groups was the training environment. The ICT group performed exercises on unstable surfaces, such as BOSU balls, Swiss balls, and wobble boards. In contrast, the TCT group conducted exercises under stable conditions, including the floor and benches.

**Table 2:** Core training exercises for ICT and TCT intervention

Core Exercises		Description of The Exercise*
Static	Prone Plank	The participant lies face down on the floor with their forearms positioned directly under the shoulders; elbows bent at a 90-degree angle. Lift the body off the ground, supporting yourself with your forearms on the BOSU ball or floor and toes on the floor, forming a straight line from head to heels. Engage the core muscles by drawing the navel toward the spine, keeping the hips level to avoid sagging or arching, and maintaining a neutral spine throughout the exercise. Avoid shrugging the shoulders or holding your breath, and hold the position with proper form for the prescribed duration.
	Shoulder Bridge	The participant lies on their back with legs extended, their heels on a Swiss ball or bench, and their hips wide apart. Arms are positioned by the sides with palms facing down. Engage the back and gluteal core muscles, then lift the hips off the ground until the body forms a straight line from the shoulders to the heels. Avoid arching the lower back or flaring the ribs. Keep the weight evenly distributed across the shoulders and heels, maintaining a stable position without allowing the hips to tilt. Maintain a neutral spine throughout the exercise and hold the position for the prescribed duration with proper form.
	Lateral Bridge (both sides)	The participant starts by lying on one side with their legs extended and stacked on top of each other, with the same side-lying foot on the floor. Place the forearm on Wobble boards or the floor directly beneath the shoulder, and lift the hips off the ground, forming a straight line from the head to the feet. Engage the core and glute muscles to maintain stability, ensuring that the body remains in a straight, plank-like position. Avoid letting the hips sag or the shoulders rotate forward. Keep the neck neutral and maintain a strong, stable posture throughout the duration of the exercise.
Dynamic	Crunch	The participant begins by lying on a Swiss ball or floor with knees bent and feet flat on the floor, hip-width apart. Place the hands lightly behind the head, ensuring the elbows are open and not pulled forward. Engage the core by drawing the navel towards the spine, then lift the head, neck, and shoulders off the ground in a controlled manner, leading with the chest. Avoid excessive pulling on the neck or tucking the chin. Pause briefly at the top of the movement, then slowly lower back to the starting position while maintaining core engagement. Repeat the movement for the prescribed number of repetitions.
	Back Extension	The participant lies face down on a Swiss ball or the floor, with the toes on the floor for stability. Place the hands lightly behind the head, keeping the elbows open. Slowly lift the upper body by extending through the lower back, raising the chest off the surface. Avoid overextending the spine or using momentum. Pause briefly at the top of the movement, ensuring the body forms a straight line from the head to the hips. Lower back down to the starting position in a controlled manner. Repeat for the prescribed number of repetitions.
	Russia Twist	The participant sits on a BOSU ball or floor with knees bent and feet lifted off the floor, hip-width apart. Lean slightly back so that the torso forms a 45-degree angle with the floor, keeping the back straight. Hold a 2.5kg weight plate or clasp hands together in front of the chest. Rotate the torso to one side and touch the weight plate to the ground on the side of the body while keeping the core engaged and maintaining controlled movement. Return to the center, then rotate to the opposite side. Avoid rounding the back or relying on momentum; instead, focus on using the core to control the motion. Repeat for the prescribed number of repetitions.

* This exercise sequence was only conducted by the *ICT* and *TCT* groups, whereas participants of the *ICT* group performed on unstable surfaces (i.e., BOSU balls, Swiss balls, and Wobble boards) and the *TCT* group performed the same exercises on stable surfaces (i.e., floor, bench).

The training protocol consisted of three sessions per week, each lasting 60 min, over the 12-week intervention period. Training intensity progressively increased across the three phases, transitioning from low intensity in the primary phase to moderate in the intermediate phase and high in the advanced phase. The progression of intensity was achieved by gradually increasing exercise duration for static core exercises and repetitions for dynamic core exercises. For instance, in the Prone Plank, Shoulder Bridge, and Lateral Bridge, the duration increased from 15–20 s in the primary phase to 35–40 s in the intermediate phase and 55–60 s in the advanced phase. Similarly, for dynamic exercises such as Crunch, Back Extension, and Russian Twist, repetitions increased from 15–20 in the primary phase to 35–40 in the intermediate phase and 55–60 in the advanced phase (see Table 3). Furthermore, all static or dynamic core training exercises were completed in 3 sets, and participants had 60–90 s rest periods between each set. Prior to and following the training sessions, the participants had 5–10 min for a light general active warm-up and cool down, which involved stretching for the upper and lower limbs, as well as core muscle groups. The details of the design of the ICT and TCT intervention program are shown in Table 3 (see Table 3).

**Table 3:** Training routine for the three phases of ICT vs TCT groups (Week 1–12)

Week	Time&Frequency	Core Training Exercises		Duration & Repetitions	Sets	Rest (s)	Intensity	
		EG/ICT	CG/TCT					
Week 1–4	1Time/5–10 min	Warm-up	Warm-up	1 rep				
	3 Times/Week 1 Hour	Prone Plank on BOSU ball	Prone Plank on the floor	15–20 s	3	60-90 s	Low-intensity	
		Shoulder Bridge on Swiss ball	Shoulder Bridge on a bench	15–20 s	3	60-90 s		
		Lateral Bridge on Wobble boards	Lateral Bridge on the floor	15–20 s	3	60-90 s		
		Crunch on a Swiss ball	Crunch on the floor	15–20 reps	3	60-90 s		
		Back Extension on Swiss ball	Back Extension on the floor	15–20 reps	3	60-90 s		
		Russia's twist on the BOSU ball	Russia's twist on the floor	15–20 reps	3	60-90 s		
	1Time/5–10 min	Relax	Relax	1 rep				
Week 5–8	1Time/5–10 min	Warm-up	Warm-up	1 rep				
	3 Times/Week 1 Hour	Prone Plank on BOSU ball	Prone Plank on the floor	35–40 s	3	60-90 s	Moderate-intensity	
		Shoulder Bridge on Swiss ball	Shoulder Bridge on a bench	35–40 s	3	60-90 s		
		Lateral Bridge on Wobble boards	Lateral Bridge on the floor	35–40 s	3	60-90 s		
		Crunch on a Swiss ball	Crunch on the floor	35–40 reps	3	60-90 s		
		Back Extension on Swiss ball	Back Extension on the floor	35–40 reps	3	60-90 s		
		Russia's twist on the BOSU ball	Russia's twist on the floor	35–40 reps	3	60-90 s		
	1Time/5–10 min	Relax	Relax	1 rep				
Week 9–12	1Time/5–10 min	Warm-up	Warm-up	1 rep				
	3 Times/Week 1 Hour	Prone Plank on BOSU ball	Prone Plank on the floor	55–60 s	3	60-90 s	High-intensity	
		Shoulder Bridge on Swiss ball	Shoulder Bridge on a bench	55–60 s	3	60-90 s		
		Lateral Bridge on Wobble boards	Lateral Bridge on the floor	55–60 s	3	60-90 s		
		Crunch on a Swiss ball	Crunch on the floor	55–60 reps	3	60-90 s		
		Back Extension on Swiss ball	Back Extension on the floor	55–60 reps	3	60-90 s		
		Russia's twist on the BOSU ball	Russia's twist on the floor	55–60 reps	3	60-90 s		
	1Time/5–10 min	Relax	Relax	1 rep				

This exercise sequence was conducted by the instability core training, *ICT* group on unstable surfaces (i.e., BOSU ball, Swiss ball, and Wobble boards) and traditional core training, *TCT* group on stable surfaces (i.e., floor, bench), whereas participants two training groups performed the same core training exercise.

To ensure consistency and standardization of exercise execution across participants, all training sessions were conducted under the direct supervision of certified strength and conditioning specialists with prior experience in core training interventions. These professionals monitored participants’ posture, technique, and adherence to the prescribed exercise parameters (duration, repetitions, rest intervals) in real time, providing immediate feedback and corrections when necessary. Before the start of the 12-week intervention, all participants underwent a 2-day familiarization and technique standardization workshop, during which correct form and movement execution for all six core exercises were demonstrated and practiced under supervision. During the intervention period, a standardized protocol checklist was used by instructors to evaluate movement quality and ensure that each exercise was performed according to defined biomechanical standards (e.g., neutral spine alignment during planks and bridges, controlled tempo during dynamic movements). This approach minimized inter-individual variation in exercise performance and ensured that any improvements in outcomes could be attributed to the training condition rather than inconsistencies in technique.

### Evaluation

Firstly, the Flamingo Balance Test (FBT) evaluated static balance ability in young male Chinese kayakers. This test required specific instruments, including a stopwatch and a metal beam measuring 50 cm in length, 5 cm in height, and 3 cm in width, with both ends supported by brackets and an anti-skid surface. Participants were instructed and warmed up before the test. During the assessment, subjects stood barefoot on the metal beam, initially holding the tester’s hand for balance. After achieving balance on their preferred leg, they bent the free leg at the knee, positioning the foot near the hip with the opposite hand. Once the tester released their hand, the stopwatch was started. Each time the participant lost balance—either by stepping off the beam or releasing the held foot—the tester stopped the stopwatch and recorded the number of balance losses, referred to as “fall mistakes.” The test lasted 60 s, with the total number of fall mistakes determining the final score, which was capped at 15 if falls exceeded this threshold [68].

Secondly, the Star Excursion Balance Test (SEBT), on the other hand, measured dynamic balance ability. This test requires a flat, non-slip surface, a tape measure, and four 120 cm marking tapes arranged at 45° angles to create eight movement directions: anterior (ANT), anterolateral (ALAT), lateral (LAT), posterolateral (PLAT), posterior (POST), posteromedial (PMED), medial (MED), and anteromedial (AMED) [69]. After a general warm-up, participants stood barefoot at the intersection of the marking tapes with their hands on their hips. They maintained a stable stance on one foot while extending the opposite foot to gently touch each directional line with their toes, returning to the starting position while keeping the supporting foot flat on the ground. The tester recorded the reach distance to the nearest 0.5 cm, and three trials were performed for each foot. The test was completed within five minutes, with the average reach distance for each direction calculated and the relative distance (%) using the formula: (average reach distance/leg length) × 100. The trial was invalid if the participant failed to return to the starting position or lost balance. Both left and right legs were tested, resulting in a total of 16 data points, with higher overall values indicating superior dynamic balance ability.

The kayaking flatwater sprint paddling performance is typically divided into two key phases, water and aerial, and four sub-phases: catch/entry, drive/pull, exit, and recovery/aerial [7]. In the paddling phases, the recovery/aerial phase refers to the stage in which the paddle is in the air, without acting on water and generating any propulsion or resistance. The other three stages are the paddle and water interaction stage, which generates power to propel the kayak forward due to the interaction between the paddle blades and water [2]. The K-1 200 m land dynamometer/ergometer sprint performance test is conducted to assess paddling performance specific to any particular phase.

Specifically, the paddling performance of stroke power and stroke rate was designed to assess the'K-1 200 m land dynamometer/ergometer sprint performance test of young Chinese male kayakers. This test utilized the Dansprint PRO Kayak dynamometer/ergometer, a specialized device manufactured in Denmark, and was conducted in a kayak-specific land dynamometer/ergometer sprint performance testing room of the Nanchang Yao Lake kayaking training base. Prior to the test, the tester configured the necessary parameters, including the kayak model, land performance type, wind resistance of the dynamometer, and basic athlete data such as height and weight. Participants were given time to warm up by running and performing light stretching exercises, including a 5–10 min light general warm-up activity and light kayaking sport-specific activity, such as jogging, stretching of limbs and core area, and specialized paddling activities. Once prepared, they seated themselves on the dynamometer cushion with their feet securely fixed on the pedals and grasped the simulated paddle with both hands. Upon receiving the command, participants were instructed to perform the test to the best of their sprint ability. The test involved completing a 200-m simulation on the land dynamometer, with the LCD digital display screen of the device automatically recording the stroke power and stroke rate parameters to determine performance. The final score was based on the recorded time displayed on the LCD screen. Further details on the procedure of the men’s K-1 200 m dynamometer sprint performance tests can be found on the official website (https://dansprint.com/vare/dansprint-pro-kayak-ergometer/).

### Statistics

Data analysis for this study was conducted using SPSS 28.0 software. Descriptive statistics, including means and standard deviations (SD), were used to report the baseline values of pre- and post-test data for balance ability and paddling performance variables. Inferential statistics were employed to compare the dependent variables of pre- and post-test measures, both within groups and between groups. The assumptions of normality and homogeneity of variances were assessed through skewness, kurtosis tests, and Levene's test. To evaluate the effects of the intervention, multivariate analysis of variance (MANOVA) was conducted using a 2 × 2 design. Effect size was calculated based on standard criteria, with η^2^ values of 0.01, 0.06, and 0.14 representing small, medium, and large effects, respectively [70]. Statistical significance was set at *P* < 0.05.

## Results

Table 4 compares balance ability and paddling performance between instability core training (ICT) and traditional core training (TCT). While both training methods led to significant improvements over the 12-week intervention, ICT demonstrated greater effectiveness for all balance ability and paddling performance variables than TCT among young male Chinese kayakers. Table 5 is the pairwise comparisons of MANOVA for balance and paddling performance variables among post-test between ICT and TCT groups. (see Tables 4 and 5).

**Table 4:** The analysis of Manova for within and between group effects of balance ability and paddling performance variables for ICT and TCT Groups

Variables		Test Time	Measurement Outcomes		Between-group Effect	Within-group Effect	
			ICT(M ± SD)	TCT (M ± SD)	ICT vs TCT*F*, *P* (*η*^2^)	ICT (T0 vs T12)*F*, *P* (*η*^2^)	TCT (T0 vs T12) *F*, *P* (*η*^2^)
Balance Ability	FBT	T0	6.60±1.77	6.83±2.26	*F* = .198, *P* = .658 (.003)	F = 68.295 *P*< .001**(.541)	*F* = 6.787 *P* = 0.012* (.105)
		T12	2.96±1.62	5.40±1.99	*F* = 26.807, *P* < .001** (.316)		
	SEBT	T0	100.51±6.90	99.54±5.92	*F* = .342, *P* = .561 (.006)	*F* = 51.753 *P* < .001**(.472)	*F* = 18.863 *P* < .001** (.249)
		T12	113.51±7.09	106.11±5.78	*F* = 19.654, *P* < .001** (.253)		
Paddling	SP	T0	261.23±4.65	259.23±4.72	*F* = 2.730, *P* = .104 (.045)	*F* = 278.796	*F* = 1917.729
		T12	328.33±21.51	318.10±5.64	*F* = 6.350, *P*= .015* (.099)	*P *< .001**(.828)	*P* < .001** (.971)
Performance	SR	T0	110.19±6.03	108.25±5.79	*F* = 1.619, *P* = .208(.027)	*F* = 42.038 *P*< .001**(.420)	*F* = 11.345 *P* = .001** (.164)
		T12	120.33±6.07	113.30±5.81	*F* = 20.9, *P*< .001** (.266)		

*ICT* instability core training, *TCT* traditional core training, *FBT* Flamingo Balance Test, *SEBT* Star Excursion Balance Test, *SP* Stroke Power, *SR* Stroke Rate, *T0* pre-intervention test, T12 12-week post-intervention test, *M* mean, *SD* standard deviation, *P* *P* value, *η*² effect size*, the mean difference is significant at the 0.05 level; **, the mean difference is significant at the 0.01 level

**Table 5:** Pairwise Comparisons of MANOVA for Balance and Paddling Performance Variables among Post-test of Between-Group Effect for ICT versus TCT Groups

Variables		(I) Group	(J) Group	Mean Difference (I-J)	Std. Error	*P* (sig)	95% Confidence Interval	
							Lower Bound	Upper Bound
Balance Ability	FBT	ICT	TCT	-2.433*	0.470	0.000 **	-3.374	-1.493
		TCT	ICT	2.433*	0.470	0.000 **	1.493	3.374
	SEBT	ICT	TCT	7.409*	1.671	0.000 **	4.064	10.754
		TCT	ICT	-7.409*	1.671	0.000 **	-10.754	-4.064
PaddlingPerformance	SP	ICT	TCT	10.233*	4.061	0.015 *	2.104	18.362
		TCT	ICT	-10.233*	4.061	0.015 *	-18.362	-2.104
	SR	ICT	TCT	7.033*	1.535	0.000 **	3.960	10.107
		TCT	ICT	-7.033*	1.535	0.000 **	-10.107	-3.960

*ICT* instability core training, *TCT* traditional core training, *FBT* Flamingo Balance Test, *SEBT* Star Excursion Balance Test, *SP* Stroke Power, *SR* troke Rate, *M* mean, *SD* standard deviation, *P* *P* value *, the mean difference is significant at the 0.05 level; **, the mean difference is significant at the 0.01 level.

In terms of static and dynamic balance ability, both groups exhibited significant time effects from the pre-test (T0) to the post-test (T12) in the Flamingo Balance Test (FBT) and Star Excursion Balance Test (SEBT), including static balance ability (*F* = 68.295, *p* < 0.001, *η*^2^ = 0.541) and dynamic balance ability (*F* = 51.753, *p* < 0.001, *η*^2^ = 0.472) in ICT group, static balance ability (*F* = 6.787, *p* = 0.012, *η*^2^ = 0.105) and dynamic balance ability (*F* = 18.863, *p* < 0.001, *η*^2^ = 0.249) in TCT group of within-group effect among young male Chinese kayakers. However, the ICT group exhibited no significant difference for the pre-test (T0) as evidenced by smaller effect sizes for static balance ability (*F* = 0.198, *p* = 0.685, *η*^2^ = 0.003) and dynamic balance ability (*F* = 0.342, *p* = 0.561, *η*^2^ = 0.006), and more pronounced enhancements for the post-test (T12) as evidenced by larger effect sizes for static balance ability (*F* = 26.807, *p* < 0.001, *η*^2^ = 0.316) and dynamic balance ability (*F* = 19.654, *p* < 0.001, *η*^2^ = 0.253), compared to the TCT group, indicating the superior efficacy of ICT in enhancing balance ability of between-group effect among young male Chinese kayakers. (see Table 4).

Similarly, both groups significantly improved the Men’s K-1 200 m land dynamometer sprint performance test from T0 to T12 for paddling performance variables, including average stroke power (*F* = 278.796, *p* < 0.001, *η*^2^ = 0.828) and stroke rate (*F* = 1917.729, *p* < 0.001, *η*^2^ = 0.971) in ICT group, average stroke power (*F* = 42.038, *p* < 0.001, *η*^2^ = 0.420) and stroke rate (*F* = 11.345, *p* < 0.001, *η*^2^ = 0.164) in ICT group of within-group effect among young male Chinese kayakers. However, the ICT group exhibited no significant difference for the pre-test (T0) as evidenced by smaller effect sizes for average stroke power (*F* = 2.730, *p* = 0.104, *η*^2^ = 0.045) and stroke rate (*F* = 1.619, *p* = 0.208, *η*^2^ = 0.027), and more pronounced enhancements for the post-test (T12) as evidenced by larger effect sizes for average stroke power (*F* = 6.350, *p* = 0.015, *η*^2^ = 0.099) and stroke rate (*F* = 20.9, *p* < 0.001, *η*^2^ = 0.266), compared to the TCT group, indicating the superior efficacy of ICT in enhancing paddling performance of between-group effect among young male Chinese kayakers. (see Table 4).

In addition to statistical significance, the reported effect sizes (*η*^2^) provide insight into the practical significance of the findings. According to Byrne’s (2013) conventional benchmarks, *η*^2^ values of 0.01, 0.06, and 0.14 represent small, medium, and large effects, respectively [70]. In this study, most post-test *η*^2^ values for the ICT group—particularly for stroke power (*η*^2^ = 0.099), stroke rate (*η*^2^ = 0.266) static balance (*η*^2^ = 0.316), and dynamic balance (*η*^2^ = 0.253)—fall within the medium-to-large effect range, indicating that the improvements are not only statistically significant but also practically meaningful in real-world athletic contexts. Comparatively, effect sizes in the TCT group, while significant, were generally smaller, suggesting ICT is more impactful for enhancing performance-critical attributes. These medium-to-large effect sizes reflect substantial improvements that are likely to translate into better kayaking performance during competition, highlighting the practical utility of incorporating ICT into training programs.

## Discussion

### Effect of ICT vs TCT on balance ability

The observed improvements in both static and dynamic balance ability among kayakers following the instability core training (ICT) intervention highlight its effectiveness in enhancing neuromuscular control, proprioception, and spinal stability—critical components for maintaining equilibrium in the athlete-paddle-boat system. ICT likely facilitated greater activation of deep stabilizing muscles, such as the transverse abdominis and multifidus, which play a crucial role in postural control and spinal stability [23]. These adaptations are particularly relevant in kayaking, where continuous postural adjustments are required to counteract external perturbations from water resistance and maintain balance during dynamic paddling movements [22]. The use of unstable surfaces, including BOSU balls, Swiss balls, and Wobble boards, in the ICT program may have contributed to enhanced proprioceptive feedback and intermuscular coordination between global and local core muscle systems, improving both static and dynamic balance [8]. The experimental group exhibited significantly more significant gains in dynamic balance ability, as reflected in the Star Excursion Balance Test (SEBT), suggesting that ICT-induced neuromuscular adaptations improved anticipatory postural adjustments and compensatory responses to destabilizing forces, which are essential for efficient force transfer and stroke execution in sprint kayaking [5]. Furthermore, the enhanced static balance ability observed in the Flamingo Balance Test (FBT) suggests that ICT promoted more excellent postural stability and trunk control by strengthening the deep core muscles responsible for stabilizing the spine under minimal external movement demands [71]. Unlike traditional core training (TCT), which primarily targets large superficial core muscles on stable surfaces, ICT engages a broader range of stabilizing and dynamic control mechanisms, optimizing motor unit recruitment and enhancing neuromuscular efficiency in sport-specific contexts [72].

The superior balance improvements observed in the ICT group reinforce previous findings that unstable surface training elicits greater activation of sensorimotor pathways and enhances spinal reflex responses, ultimately improving an athlete’s ability to maintain balance under dynamic and unpredictable conditions [73]. In contrast, TCT, with its emphasis on isotonic contractions and stable support, may not sufficiently challenge the proprioceptive and neuromuscular systems required for high-level balance control in kayaking, potentially limiting its effectiveness in improving balance performance under real-world competitive conditions [74]. Given the pivotal role of balance in optimizing stroke mechanics, maintaining an erect posture, and minimizing unnecessary energy expenditure during kayaking, these findings underscore the necessity of incorporating instability-based core training into the preparatory regimens of young male Chinese kayakers to enhance both static and dynamic balance ability, ultimately contributing to improved stroke power and stroke rate for paddling performance [2]. However, it is acknowledged that part of the observed improvements, particularly in both groups, may be partially influenced by learning effects from repeated testing or placebo responses due to increased training attention. Future studies should include familiarization sessions and placebo-controlled designs to more precisely isolate the effects of ICT interventions from such confounding variables.

While the current study focused on kayaking, insights from related sports such as swimming and rowing offer valuable comparative perspectives on core training strategies. In competitive swimming, instability-based core training has been shown to improve stroke efficiency and streamline posture by enhancing trunk stability and body alignment under fluid resistance conditions [75]. Similarly, in rowing, core training programs incorporating dynamic balance elements have been linked to improved stroke symmetry and reduced lower back injury risk due to better neuromuscular control and load distribution [76]. These findings support the idea that instability-oriented core training enhances sport-specific postural control and force transfer across various aquatic and cyclic sports. Unlike traditional core training modalities, which often rely on stable, linear movements, ICT emphasizes reactive stability and multidirectional coordination—capabilities that are essential for managing the unpredictable perturbations encountered in water-based sports. Therefore, integrating evidence from swimming and rowing not only reinforces the applicability of ICT in kayaking but also highlights the broader relevance of instability-based training for optimizing balance-related performance outcomes across disciplines with similar biomechanical and neuromuscular demands.

### Effect of ICT vs TCT on paddling performance

The improvements in average stroke power and stroke rate observed in the Men’s K-1 200 m land dynamometer sprint performance test following the ICT intervention suggest that enhanced static and dynamic balance ability contribute to more efficient force generation and paddling parameters. Static balance ability is essential for maintaining an optimal trunk posture, minimizing excessive torso movement, and ensuring efficient force transmission from the lower body to the upper limbs during each stroke cycle, thereby maximizing stroke power output [63, 77]. Additionally, dynamic balance ability is pivotal in facilitating rotational torque and trunk flexion–extension movements, which are critical for increasing stroke rate and optimizing stroke efficiency in sprint kayaking [18, 78]. The 12-week ICT program likely enhanced neuromuscular coordination and proprioceptive control, leading to improved motor unit synchronization and faster recruitment of core musculature during high-intensity paddling efforts. The activation of deep stabilizing muscles, such as the transverse abdominis and multifidus, reinforced spinal stability and minimized energy dissipation, allowing for more powerful and frequent stroke execution [5, 23]. Unlike TCT, which primarily emphasizes isotonic contractions of superficial balance ability under stable conditions, ICT promotes dynamic core activation by incorporating unstable surfaces, thereby improving sensorimotor integration and intermuscular coordination between trunk and limb muscles [30, 79, 80]. These adaptations are particularly advantageous in sprint kayaking, where precise and rapid trunk adjustments are required to maintain balance and optimize propulsion efficiency [22].

Our findings align with previous research supporting the efficacy of instability core training in improving athletic performance. For instance, Granacher et al. (2013) and Hammami et al. (2023) reported that instability training improves trunk muscle activation and balance control, which in turn enhances sport-specific movement efficiency for athletes [34, 48]. Similarly, Brown et al. (2023) demonstrated that dynamic balance training increased core muscle co-contraction and improved postural control in athletes, reinforcing the potential benefit of ICT for the paddling performance of kayakers [5]. However, contradictory findings have also been reported. For example, Parkhouse & Ball. (2011) and Sanghvi et al. (2014) found no significant improvements in athletic performance following instability core training, suggesting that ICT may not directly translate to enhanced sport-specific outputs [35, 81]. The inconsistency may stem from differences in training protocols, athlete populations, or performance assessment methods. Notably, some previous studies employed general or non-specific core training interventions or assessed outcomes using non-functional tasks, which may limit the transference to sport-specific contexts such as sprint kayaking. In contrast, our study applied a targeted ICT protocol emphasizing balance-challenging conditions and sport-relevant movement patterns, likely contributing to the observed improvements.

These findings reinforce the importance of sport-specific instability core training (ICT) in enhancing balance ability and paddling performance by improving both stroke power and stroke rate. ICT exercises involving unstable surfaces (e.g., Swiss balls, BOSU balls, or balance boards) should be prioritized by coaches and sports scientists to develop neuromuscular efficiency, spinal stability, and dynamic core control. Such training not only improves paddling-specific performance but may also contribute to injury prevention by enhancing postural control and core endurance. To optimize practical applications, ICT should be designed to closely replicate sport-specific demands and movement patterns in kayaking. For future research, it is recommended to investigate the long-term effects of ICT on competition-level performance, explore the optimal balance between instability and resistance load in core training, and incorporate electromyographic (EMG) assessments to validate neuromuscular activation mechanisms. Additionally, examining sex-based and age-related differences in responsiveness to ICT will provide further insights into individualized program design for sprint kayakers.

In summary for this section, based on these findings, coaches and practitioners are encouraged to incorporate ICT into the regular training programs of sprint kayakers, especially during the preparatory phase of the season. Specifically, exercises involving unstable surfaces (e.g., Swiss balls, BOSU balls, balance boards) should be used to target dynamic trunk stability, neuromuscular coordination, and proprioceptive control. These drills can be integrated 2–3 times per week as part of strength or technical sessions. Additionally, ICT protocols should progressively simulate sport-specific postures and movement patterns to maximize transfer to paddling performance and reduce the risk of injury through improved postural control and core endurance.

## Limitations

This study has several limitations that warrant consideration. First, the ICT intervention was limited to Wobble boards, Swiss balls, and BOSU balls as unstable surfaces. Since different unstable environments may produce varying training effects, future research should explore additional options, such as elastic bands, foam rollers, suspension chains, and natural surfaces like sand and water, to optimize ICT effectiveness. Second, the study assessed the paddling performance of stroke power and stroke rate improvements based on dynamometer sprint performance on land, which may not fully reflect on-water sprint performance. Future studies should extend the evaluation to kayak-specific water sprinting to enhance practical relevance. Third, further research should examine the impact of ICT on athletes for more diverse samples and long training intervention duration of different genders, ages, and training levels, as well as athletes from other water-based sports, including sailing, swimming, and rowing. Another limitation of this study is that all performance assessments were conducted in a land-based setting using a kayak ergometer, which may not fully replicate the dynamic and unstable conditions of actual on-water sprint kayaking. Although land-based tests offer controlled and repeatable conditions, they may limit the ecological validity of the findings. Future studies are encouraged to incorporate on-water performance assessments to better capture sport-specific movement patterns and enhance the applicability of results to real-world paddling performance. Therefore, expanding research in these areas will help establish whether ICT is superior to TCT in improving balance ability and paddling performance, providing broader validation of its applicability across diverse athletic populations.

## Conclusion

In conclusion, after a 12-week intervention, both TCT and ICT led to significant improvements in balance ability and paddling performance. However, ICT proved to be more effective than TCT, particularly in enhancing static and dynamic balance ability, as well as key paddling metrics such as stroke power and stroke rate among young male kayakers in Jiangxi province, China. These findings add to the growing body of research highlighting the importance of tailored training approaches in sports and offer a solid foundation for future studies exploring the role of instability-based training in balance and sprint performance development.

## Clinical trial number

Trial registration: Current controlled trials permission of protocol in ClinicalTrials.gov PRS (https://clinicaltrials.gov/) (NCT06432595). In addition, this study was conducted and reported in accordance with the CONSORT (Consolidated Standards of Reporting Trials) guidelines.

## Informed consent

All the kayakers voluntarily participated in the study and provided informed consent.

## Data Availability

The datasets generated during and/or analyzed during the current study are available from the corresponding author on reasonable request.

## Abbreviations

ICT: Instability Core Training
TCT: Traditional Core Training
FBT: Flamingo Balance Test
SEBT: Star Excursion Balance Test
SP: Stroke Power
SR: Stroke Rate
η^2^: Eta Square (Effect Size)
RCT: Randomized Controlled Trial
MANOVA: Multivariate Analysis of Variance

## References

[1] Harrison SM, Cleary PW, Cohen RCZ. Dynamic simulation of flatwater kayaking using a coupled biomechanical-smoothed particle hydrodynamics model. Hum Mov Sci. 2019;64:252–73. 10.1016/j.humov.2019.02.003. 30822692 10.1016/j.humov.2019.02.003

[2] Prétot C, Carmigniani R, Hasbroucq L, Labbé R, Boucher J-P, Clanet C. On the physics of kayaking. Appl Sci. 2022;12:1–14. 10.3390/app12188925.

[3] McDonnell LK, Hume PA, Nolte V. An observational model for biomechanical assessment of sprint kayaking technique. Sports Biomech. 2012;11:507–23. 10.1080/14763141.2012.724701. 23259240 10.1080/14763141.2012.724701

[4] Bertozzi F, Porcelli S, Marzorati M, Pilotto AM, Galli M, Sforza C, et al. Whole-body kinematics during a simulated sprint in flat-water kayakers. Eur J Sport Sci. 2022;22:817–25. 10.1080/17461391.2021.1930190. 33980124 10.1080/17461391.2021.1930190

[5] Brown MB, Peters R, Lauder MA. Contribution of trunk rotation and abdominal muscles to sprint kayak performance. J Hum Kinet. 2023;90:5–15. 10.5114/jhk/169939. 38380295 10.5114/jhk/169939 PMC10875689

[6] López-Plaza D, Alacid F, Muyor JM, López-Miñarro PÁ. Sprint kayaking and canoeing performance prediction based on the relationship between maturity status, anthropometry and physical fitness in young elite paddlers. J Sports Sci. 2017;35:1083–90. 10.1080/02640414.2016.1210817. 27433884 10.1080/02640414.2016.1210817

[7] McDonnell LK, Hume PA, Nolte V. A deterministic model based on evidence for the associations between kinematic variables and sprint kayak performance. Sports Biomech. 2013;12:205–20. 10.1080/14763141.2012.760106. 24245047 10.1080/14763141.2012.760106

[8] Gomes BB, Ramos NV, Conceição F, Sanders R, Vaz M, Vilas-Boas JP. Paddling time parameters and paddling efficiency with the increase in stroke rate in kayaking. Sports Biomech. 2022;21:1303–11. 10.1080/14763141.2020.1789204. 32727291 10.1080/14763141.2020.1789204

[9] Begon M, Colloud F, Sardain P. Lower limb contribution in kayak performance: modelling, simulation and analysis. Multibody Syst Dyn. 2010;23:387–400. 10.1007/s11044-010-9189-8.

[10] Gomes BB, Ramos NV, Conceição FAV, Sanders RH, Vaz MAP, Vilas-Boas JP. Paddling force profiles at different stroke rates in elite sprint kayaking. J Appl Biomech. 2015;31:258–63. 10.1123/jab.2014-0114. 25838207 10.1123/jab.2014-0114

[11] Loturco I, Pereira LA, Moura TBMA, McGuigan MR, Boullosa D. Effects of different conditioning activities on the sprint performance of elite sprinters: a systematic review with meta-analysis. Int J Sports Physiol Perform. 2024;19:712–21. 10.1123/ijspp.2024-0005. 38823792 10.1123/ijspp.2024-0005

[12] Wei H, Ge G, Colbourn CJ. The existence of well-balanced triple systems. J Comb Des. 2016;24:77–100. 10.1002/jcd.21508.

[13] Tayshete I, Akre M, Ladgaonkar S, Kumar A. Comparison of the effect of proprioceptive training and core muscle strengthening on the balance ability of adolescent taekwondo athletes. Int J Health Sci Res. 2020;10:268–79.

[14] Tian M. Theories of sport training. 3rd ed. Beijing: Chinese Higher Education Press; 2006.

[15] Deng S, Wang J, Qiao D, Hao X. Exercise physiology textbook for sport major college students. Beijing: China Higher Education Press; 2018.

[16] Davidek P, Andel R, Kobesova A. Influence of dynamic neuromuscular stabilization approach on maximum kayak paddling force. J Hum Kinet. 2018;61:15–27. 10.1515/hukin-2017-0127. 29599856 10.1515/hukin-2017-0127 PMC5873333

[17] Lum D, Barbosa TM, Balasekaran G. Sprint kayaking performance enhancement by isometric strength training inclusion: a randomized controlled trial. Sports. 2021;9:1–12. 10.3390/sports9020016. 10.3390/sports9020016 PMC7909782 33494230

[18] Shao X. New perspective on physical fitness training of kayak players—core stability training. Si Chuan Sports Sci. 2013;6:76–84.

[19] Abelleira-Lamela T, Vaquero-Cristóbal R, Esparza-Ros F, Marcos-Pardo PJ. Biomechanical adaptations in kayakers of different competitive levels and the relationship with the kayak elements. Appl Sci. 2020;10:1–11. 10.3390/app10238389.

[20] Edriss S, Romagnoli C, Caprioli L, Zanela A, Panichi E, Campoli F, et al. The role of emergent technologies in the dynamic and kinematic assessment of human movement in sport and clinical applications. Appl Sci. 2024;14:1–43. 10.3390/app14031012.

[21] Brown MB, Lauder M, Dyson R. Activation and contribution of trunk and leg musculature to force production during on-water sprint kayak performance. Proc 28th Int Conf Biomech Sport. 2010;1:203–6.

[22] Bjerkefors A, Tarassova O, Rosén JS, Zakaria P, Arndt A. Three-dimensional kinematic analysis and power output of elite flat-water kayakers. Sports Biomech. 2018;17:414–27. 10.1080/14763141.2017.1359330. 28929926 10.1080/14763141.2017.1359330

[23] Behm DG, Drinkwater EJ, Willardson JM, Cowley PM. The use of instability to train the core musculature. Appl Physiol Nutr Metab. 2010;35:91–108. 10.1139/H09-127. 20130672 10.1139/H09-127

[24] Zemková E. Science and practice of core stability and strength testing. Phys Act Rev. 2018;6:181–93. 10.16926/par.2018.06.23.

[25] Gao J, Fu X, Xu H, Guo Q, Wang X. The effect of instability resistance training on balance ability among athletes: a systematic review. Front Physiol. 2025;15:1–14. 10.3389/fphys.2024.1434918. 10.3389/fphys.2024.1434918 PMC11746901 39839523

[26] Granacher U, Schellbach J, Klein K, Prieske O, Baeyens J-P, Muehlbauer T. Effects of core strength training using stable versus unstable surfaces on physical fitness in adolescents: a randomized controlled trial. BMC Sports Sci Med Rehabil. 2014;6:1–11. 10.1186/2052-1847-6-40. 25584193 10.1186/2052-1847-6-40 PMC4290805

[27] Hsu S-L, Oda H, Shirahata S, Watanabe M, Sasaki M. Effects of core strength training on core stability. J Phys Ther Sci. 2018;30:1014–8. 10.1589/jpts.30.1014. 30154592 10.1589/jpts.30.1014 PMC6110226

[28] Oliva-Lozano JM, Muyor JM. Core muscle activity during physical fitness exercises: a systematic review. Int J Environ Res Public Health. 2020;17:1–38. 10.3390/ijerph17124306. 10.3390/ijerph17124306 PMC7345922 32560185

[29] Lee S-J, Kim Y-N, Lee D-K. The effect of flexi-bar exercise with vibration on trunk muscle thickness and balance in university students in their twenties. J Phys Ther Sci. 2016;28:1298–302. 10.1589/jpts.28.1298. 27190471 10.1589/jpts.28.1298 PMC4868231

[30] Xu J, Zeng J, Wu R, Wang G, Ma G, Xu F. The interpretation, structural function, and application analysis of instability resistance training. J Harbin Sport Univ. 2020;38:78–85.

[31] Behm DG, Wahl MJ, Button DC, Power KE, Anderson KG. Relationship between hockey skating speed and selected performance measures. J Strength Cond Res. 2005;19:326–31. 15903370 10.1519/R-14043.1

[32] Borghuis AJ, Lemmink KAPM, Hof AL. Core muscle response times and postural reactions in soccer players and nonplayers. Med Sci Sports Exerc. 2011;43:108–14. 10.1249/MSS.0b013e3181e93492. 20508535 10.1249/MSS.0b013e3181e93492

[33] Cuğ M, Ak E, Özdemir RA, Korkusuz F, Behm DG. The effect of instability training on knee joint proprioception and core strength. J Sports Sci Med. 2012;11:468–74. 24149355 PMC3737939

[34] Granacher U, Lacroix A, Muehlbauer T, Roettger K, Gollhofer A. Effects of core instability strength training on trunk muscle strength, spinal mobility, dynamic balance and functional mobility in older adults. Gerontology. 2013;59:105–13. 23108436 10.1159/000343152

[35] Sanghvi N, Dabholkar Y, Yardi S. A comparative study between stable and unstable surface training on transversus abdominis muscle and functional performance in male cricketers. Indian J Physiother Occup Ther. 2014;8:232–8. 10.5958/j.0973-5674.8.1.044.

[36] Saeterbakken AH, Olsen A, Behm DG, Bardstu HB, Andersen V. The short- and long-term effects of resistance training with different stability requirements. PLoS ONE. 2019;14:e0214302. 10.1371/journal.pone.0214302. 30934001 10.1371/journal.pone.0214302 PMC6443166

[37] Makhlouf I, Chaouachi A, Chaouachi M, Ben Othman A, Granacher U, Behm DG. Combination of agility and plyometric training provides similar training benefits as combined balance and plyometric training in young soccer players. Front Physiol. 2018;9:1611. 10.3389/fphys.2018.01611. 30483158 10.3389/fphys.2018.01611 PMC6243212

[38] Negra Y, Chaabene H, Sammoud S, Bouguezzi R, Mkaouer B, Hachana Y. Effects of plyometric training on components of physical fitness in prepuberal male soccer athletes: the role of surface instability. J Strength Cond Res. 2017;31:3295–304. 10.1519/JSC.0000000000002262. 29023331 10.1519/JSC.0000000000002262

[39] Wee EH, Fatt OT. Effects of six weeks Swiss ball training on balance and agility of college soccer players. J Soc Sci. 2019;2:26–33.

[40] Gidu DV, Badau D, Stoica M, Aron A, Focan G, Monea D. The effects of proprioceptive training on balance, strength, agility and dribbling in adolescent male soccer players. Int J Environ Res Public Health. 2022;19:2028. 10.3390/ijerph19042028. 35206215 10.3390/ijerph19042028 PMC8871985

[41] Granacher U, Prieske O, Majewski M, Büsch D, Muehlbauer T. The role of instability with plyometric training in sub-elite adolescent soccer players. Int J Sports Med. 2015;36(5):386–94. 10.1055/s-0034-1395519. 25665004 10.1055/s-0034-1395519

[42] Chaouachi M, Granacher U, Makhlouf I, Hammami R, Behm DG, Chaouachi A. Within session sequence of balance and plyometric exercises does not affect training adaptations with youth soccer athletes. J Sports Sci Med. 2017;16:125–36. 28344461 PMC5358022

[43] Thielen SP, Christensen BK, Bond CW, Hackney KJ, Moen JT. A comparison of the effects of a six-week traditional squat and suspended load squat program in collegiate baseball players on measures of athletic performance. Int J Kinesiol Sports Sci. 2020;8(4):40–51. 10.7575/aiac.ijkss.v.8n.4p51.

[44] Zemková E, Hamar D. The effect of 6-week combined agility-balance training on neuromuscular performance in basketball players. J Sports Med Phys Fitness. 2010;50(3):262–7. 20842085

[45] Hammami M, Gaamouri N, Ramirez-Campillo R, Aloui G, Shephard RJ, Hill L. Effects of supplemental jump and sprint exercise training on sand on athletic performance of male U17 handball players. Int J Sports Sci Coach. 2022;17(2):376–84. 10.1177/17479541211025731.

[46] Hammami M, Bragazzi NL, Hermassi S, Gaamouri N, Aouadi R, Shephard RJ. The effect of a sand surface on physical performance responses of junior male handball players to plyometric training. BMC Sports Sci Med Rehabil. 2020;12:26. 10.1186/s13102-020-00176-x. 32351699 10.1186/s13102-020-00176-x PMC7183654

[47] Kang SH, Kim CW, Kim YI, Kim KB, Lee SS, Shin KO. Alterations of muscular strength and left and right limb balance in weightlifters after an 8-week balance training program. J Phys Ther Sci. 2013;25(7):895–900. 10.1589/jpts.25.895. 24259879 10.1589/jpts.25.895 PMC3820381

[48] Hammami R, Nobari H, Hanen W, Gene-Morales J, Rebai H, Colado JC. Exploring of two different equated instability resistance training programs on measure of physical fitness and lower limb asymmetry in pre-pubertal weightlifters. BMC Sports Sci Med Rehabil. 2023;15:40. 10.1186/s13102-023-00652-0. 36959677 10.1186/s13102-023-00652-0 PMC10037902

[49] Oliver GD, Brezzo RD. Functional balance training in collegiate women athletes. J Strength Cond Res. 2009;23(7):2124–9. 10.1519/JSC.0b013e3181b3dd9e. 19855341 10.1519/JSC.0b013e3181b3dd9e

[50] Eisen TC, Danoff JV, Leone JE, Miller TA. The effects of multiaxial and uniaxial unstable surface balance training in college athletes. J Strength Cond Res. 2010;24(6):1740–5. 10.1519/JSC.0b013e3181e2745f. 20555272 10.1519/JSC.0b013e3181e2745f

[51] Norambuena Y, Winkler L, Guevara R, Lavados P, Monrroy M, Ramírez-Campillo R. 5-week suspension training program increase physical performance of youth judokas: a pilot study. Retos. 2021;39:137–42. 10.47197/retos.v0i39.78624.

[52] Prasetyo H, Prasetyo Y, Hartanto A. Circuit training bosu ball: effect on balance and accuracy of archery athletes. Pedagogy Phys Cult Sports. 2023;27(3):229–34. 10.15561/26649837.2023.0307.

[53] Cabrejas Mata C, Morales J, Solana-Tramunt M, Nieto Guisado A, Badiola A, Campos-Rius J. Does 8 Weeks of integrated functional core and plyometric training improve postural control performance in young rhythmic gymnasts? Mot Control. 2022;26(4):568–90. 10.1123/mc.2022-0046. 10.1123/mc.2022-0046 35894881

[54] Zhao W, Wang C, Bi Y, Chen L. Effect of integrative neuromuscular training for injury prevention and sports performance of female badminton players. Biomed Res Int. 2021;2021:5555853. 10.1155/2021/5555853. 33987438 10.1155/2021/5555853 PMC8093055

[55] Romero-Franco N, Martínez-López E, Lomas-Vega R, Hita-Contreras F, Martínez-Amat A. Effects of proprioceptive training program on core stability and center of gravity control in sprinters. J Strength Cond Res. 2012;26(8):2071–7. 10.1519/JSC.0b013e31823b06e6. 21997455 10.1519/JSC.0b013e31823b06e6

[56] Gönener U, Gönener A. How balance training on different types of surfaces effect dynamic balance ability and postural sway of gymnast children? Prog Nutr. 2020;22(Suppl 1):131–7. 10.23751/pn.v22i1-S.9806.

[57] Cumming SP, Lloyd RS, Oliver JL, Eisenmann JC, Malina RM. Bio-banding in sport: applications to competition, talent identification, and strength and conditioning of youth athletes. Strength Cond J. 2017;39(2):34–47. 10.1519/SSC.0000000000000281.

[58] Ding P. The Asian man 200m single kayak athlete oarsmanship and characteristics of competitive velocity structure. China Sport Sci Technol. 2011;47(6):69–72.

[59] Wu L, Liu F, Fang H. Technical analysis of setting sail for men’s single kayak (still water) short distance 200m event. Zhejiang Sport Sci. 2013;35(4):60–5.

[60] Ying Y. The characteristics of Chinese teenagers kayak flatwater 200 meters. J Anhui Sports Sci. 2013;34(5):35–46.

[61] He J, Yu L, Ni Z. Comparative analysis of physical fitness survey between Chinese and foreign male canoe elite athletes. Stationery Technol. 2018;15:182–3.

[62] Li Y. Comparative analysis of the strength quality of Shandong kayaking team athletes and the national team champion model. Boxing Fighting. 2022;10:70–2.

[63] Xian C. Core strength training in youth canoeing physical training application research. Sports Train. 2020;10:46–9. 10.16655/j.cnki.2095-2813.2020.07.046.

[64] Moher D, Hopewell S, Schulz KF, Montori V, Gøtzsche PC, Devereaux PJ, et al. CONSORT 2010 explanation and elaboration: Updated guidelines for reporting parallel group randomised trials. Int J Surg. 2012;10(1):28–55. 10.1016/j.ijsu.2011.10.001. 22036893 10.1016/j.ijsu.2011.10.001

[65] Gao J, Abdullah BB, Dev RDO, Guo Q, Lin X. Effect of instability resistance training on sports performance among athletes: a systematic review. Rev Psicol Deporte. 2023;32(3):292–309.

[66] Gao J, Abdullah BB, Omar Dev RD. Effect of instability resistance training on core muscle strength among athletes: a systematic review. Int J Human Mov Sports Sci. 2024;12(2):391–402. 10.13189/saj.2024.120214.

[67] Cramer H, Ward L, Steel A, Lauche R, Dobos G, Zhang Y. Prevalence, patterns, and predictors of yoga use: results of a U.S. nationally representative survey. Am J Prev Med. 2016;50(2):230–5. 10.1016/j.amepre.2015.07.037. 26497261 10.1016/j.amepre.2015.07.037

[68] Rami PV, Prabhakar MM. Comparison of static balance in male football and basketball players by using flamingo balance test. Int J Physiother. 2018;5(5):162–6. 10.15621/ijphy/2018/v5i5/177432.

[69] Plisky PJ, Rauh MJ, Kaminski TW, Underwood FB. Star excursion balance test as a predictor of lower extremity injury in high school basketball players. J Orthop Sports Phys Ther. 2006;36(12):911–9. 17193868 10.2519/jospt.2006.2244

[70] Byrne BM. Structural equation modeling with Mplus: Basic concepts, applications, and programming. New York: Routledge; 2013. 10.4324/9780203807644.

[71] Nuhmani S. Efficacy of dynamic Swiss ball training in improving the core stability of collegiate athletes. Phys Act Rev. 2021;9:9–15. 10.16926/par.2021.09.02.

[72] Dong K, Yu T, Chun B. Effects of core training on sport-specific performance of athletes: a meta-analysis of randomized controlled trials. Behav Sci. 2023;13(2):1–12. 10.3390/bs13020148. 10.3390/bs13020148 PMC9952339 36829378

[73] Fuentes-García MA, Malchrowicz-Mośko E, Castañeda-Babarro A. Effects of variable resistance training versus conventional resistance training on muscle hypertrophy: a systematic review. Sport Sci Health. 2024;20:37–45. 10.1007/s11332-023-01103-6.

[74] Giancotti GF, Fusco A, Iannaccone A, Cortis C. Short-term effects of suspension training on strength and power performances. J Funct Morphol Kinesiol. 2018;3(4):51. 10.3390/jfmk3040051. 33466980 10.3390/jfmk3040051 PMC7739337

[75] Khiyami A, Nuhmani S, Joseph R, Abualait TS, Muaidi Q. Efficacy of core training in swimming performance and neuromuscular parameters of young swimmers: a randomised control trial. J Clin Med. 2022;11(11):3198. 10.3390/jcm11113198. 35683586 10.3390/jcm11113198 PMC9181058

[76] Thiele D, Prieske O, Chaabene H, Granacher U. Effects of strength training on physical fitness and sport-specific performance in recreational, sub-elite, and elite rowers: a systematic review with meta-analysis. J Sports Sci. 2020;38(10):1186–95. 10.1080/02640414.2020.1745502. 32216524 10.1080/02640414.2020.1745502

[77] Goreham J. Investigating the determinants of sprint kayaking performance [PhD thesis]. Dalhousie University; 2023. https://dalspace.library.dal.ca/items/c016b04b-e23c-484d-a572-11601d62c034.

[78] Wu C, Cheong M, Wang Y, Wang X, Zhang Q, Li M, et al. Impact of functional training on functional movement and athletic performance in college dragon boat athletes. Int J Environ Res Public Health. 2023;20(5):1–11. 10.3390/ijerph20053897. 10.3390/ijerph20053897 PMC10002020 36900907

[79] Clark DR, Lambert MI, Hunter AM. Contemporary perspectives of core stability training for dynamic athletic performance: a survey of athletes, coaches, sports science and sports medicine practitioners. Sports Med Open. 2018;4:32. 10.1186/s40798-018-0150-3. 30014195 10.1186/s40798-018-0150-3 PMC6047949

[80] Glass SC, Wisneski KA. Effect of instability training on compensatory muscle activation during perturbation challenge in young adults. J Funct Morphol Kinesiol. 2023;8(3):136. 10.3390/jfmk8030136. 37754969 10.3390/jfmk8030136 PMC10531879

[81] Parkhouse KL, Ball N. Influence of dynamic versus static core exercises on performance in field-based fitness tests. J Bodyw Mov Ther. 2011;15(4):517–24. 10.1016/j.jbmt.2011.01.004. 21943626 10.1016/j.jbmt.2010.12.001

